# Model-based analysis of an outbreak of bubonic plague in Cairo in 1801

**DOI:** 10.1098/rsif.2017.0160

**Published:** 2017-06-21

**Authors:** Xavier Didelot, Lilith K. Whittles, Ian Hall

**Affiliations:** 1 Department of Infectious Disease Epidemiology, School of Public Health, Imperial College London, Norfolk Place, London W2 1PG, UK; 2 Bioterrorism and Emerging Disease Analysis, Emergency Response Department, Health Protection and Medical Directorate, Public Health England, Porton Down SP4 0JG, UK

**Keywords:** bubonic plague, infectious disease model, palaeoepidemiology, Bayesian analysis

## Abstract

Bubonic plague has caused three deadly pandemics in human history: from the mid-sixth to mid-eighth century, from the mid-fourteenth to the mid-eighteenth century and from the end of the nineteenth until the mid-twentieth century. Between the second and the third pandemics, plague was causing sporadic outbreaks in only a few countries in the Middle East, including Egypt. Little is known about this historical phase of plague, even though it represents the temporal, geographical and phylogenetic transition between the second and third pandemics. Here we analysed in detail an outbreak of plague that took place in Cairo in 1801, and for which epidemiological data are uniquely available thanks to the presence of medical officers accompanying the Napoleonic expedition into Egypt at that time. We propose a new stochastic model describing how bubonic plague outbreaks unfold in both rat and human populations, and perform Bayesian inference under this model using a particle Markov chain Monte Carlo. Rat carcasses were estimated to be infectious for approximately 4 days after death, which is in good agreement with local observations on the survival of infectious rat fleas. The estimated transmission rate between rats implies a basic reproduction number *R*_0_ of approximately 3, causing the collapse of the rat population in approximately 100 days. Simultaneously, the force of infection exerted by each infected rat carcass onto the human population increases progressively by more than an order of magnitude. We also considered human-to-human transmission via pneumonic plague or human specific vectors, but found this route to account for only a small fraction of cases and to be significantly below the threshold required to sustain an outbreak.

## 1. Introduction

Bubonic plague is arguably the most devastating infectious disease that mankind has ever been confronted with. Its causative agent, *Yersinia pestis*, has recently been detected in several human remains dated from the third millennium BCE but at that time lacked the ability to cause epidemics of bubonic plague transmitted by ectoparasites [1,2]. Genetic adaptation to the flea-borne pathogenic lifestyle is estimated to have happened shortly before the beginning of the first millennium BCE [1,3,4], which coincides with some of the first historical descriptions of epidemics that have been putatively attributed to bubonic plague. Ancient historical descriptions are, however, typically insufficient to reach a clear verdict of plague. For example, the disease that struck the Philistines in the eleventh century BCE has been proposed to be the earliest known outbreak of bubonic plague, but could in fact have been caused by dysentery or tularaemia [5,6]. Likewise, the outbreak that ravaged Athens in 430 BCE could have been a number of infectious diseases other than plague, such as Ebola [7,8]. Although there would certainly have been earlier cases at least sporadically, the first human deaths that have been conclusively attributed to *Y. pestis* using DNA evidence occurred in the sixth century CE, during the so-called plague of Justinian [9,10]. This epidemic was first reported by the contemporary historian Procopius in Egypt in 541 CE, and it would have spread rapidly on ships carrying grain to Constantinople and throughout the Byzantine empire [11]. The first plague pandemic followed, which ravaged Mediterranean and European regions through several waves over the next 200 years [12]. The second plague pandemic started in 1347 with the so-called Black Death epidemic. Once again, DNA evidence has been used to incriminate *Y. pestis* as the infectious agent [13,14]. The epidemic originated in Central Asia before spreading throughout Europe via trade routes [15]. In Western Europe, the second pandemic lasted until the end of the seventeenth century, with the Marseilles plague of 1720 being an exceptionally late outbreak [16]. For example, in England, the last major outbreak took place in 1665 in London and a few secondary locations [17,18]. Finally, a third pandemic started around 1855 which ravaged China and India for almost a century, and during which the causative bacteria *Y. pestis* was discovered as well as its flea-borne mode of transmission [19].

Phylogenetic evidence indicates that the third pandemic was caused by a direct descendant from the lineage that had caused the second pandemic [10,15]. Between the end of the second and the beginning of the third pandemic, this lineage would have been restricted to the Middle East [10]. Outbreaks of bubonic plague were indeed regularly reported during the interpandemic period, for example, in Iraq [20], in Syria [21] and in Egypt [21–25]. A comprehensive survey of historical reports of epidemics shows that throughout the eighteenth century and the first half of the nineteenth century, plague was documented in Asia Minor in approximately 85% of years, in Lower Egypt in approximately 30% of years and in Syria in approximately 20% of years [26]. This recurrence of plague in the Middle East at a time when Europe was free of it significantly contributed to the weakening of the Ottoman Empire [22]. Several Western visitors were surprised by the lack of measures taken by the local population to try and protect itself from the disease [25,26]. Very high plague death tolls were recorded by contemporary writers, but many of these were exaggerated and the aetiology is often unclear [25,27]. Thus, although it is beyond doubt that the Middle East was frequently hit by plague between 1700 and 1850, little is known about this important connecting link between the second and third pandemics, and there are virtually no reliable data available for formal epidemiological investigation.

A notable exception concerns the short period from 1798 to 1801 during which a French revolutionary expedition into Ottoman Egypt was led by the future emperor Napoleon Bonaparte. Many French scientists accompanied this expedition, who studied many aspects of the Egyptian country they visited, such as its topography, climate, history, demography and languages. The expedition included a large number of medical officers headed by René Desgenettes who together collected a wealth of medical observations about the health of the Egyptian population [28,29]. Upon his return, Desgenettes reported his findings in a book entitled ‘Histoire médicale de l'armée d'Orient’ which includes a table of the daily number of deaths recorded among men, women and children living in Cairo [30]. This daily mortality statistical table covers an epidemic of plague that took place in early 1801 and which is briefly mentioned in Desgenettes' book [30] as well as a few monographs written by other medical officers returning from the French expedition [31–33] and secondary sources [34–36]. These data present a unique opportunity to dissect how an outbreak of bubonic plague unfolded in interpandemic Egypt.

## 2. Material and methods

### 2.1. Data used in this study

The number of men, women and children reported dead in Cairo between 19 November 1798 and 4 July 1801 was published as a table in the book ‘Histoire médicale de l'armée d'Orient’ by René-Nicolas Dufriche Desgenettes, the chief medical officer of the Napoleonic French expedition into Egypt [30]. This book, including the table of mortality, was digitalized as part of Project Gutenberg and is available at https://www.gutenberg.org/ebooks/28249. The Ebook version of this book was produced by Mireille Harmelin, Christine P. Travers and the Online Distributed Proofreading Team at http://www.pgdp.net based on images generously made available by the Bibliothèque nationale de France (BnF/Gallica) at http://gallica.bnf.fr. Manual curation of the table in the Ebook revealed a few mistakes which were corrected and the data analysed here are contained in electronic supplementary material, table S1.

### 2.2. Mathematical model of bubonic plague outbreak

A bubonic plague model was previously proposed to explain the long-term dynamics of bubonic plague in Europe [37,38]. We modified this model with two goals in mind: firstly to analyse outbreaks on a shorter time scale and secondly to avoid the use of parameters and processes with high uncertainty attached. The new model contains three states for the rat population (*S*_R_, *I*_R_ and *Q*), three states for the human populations (*S*_H_, *I*_H_, *D*_H_) and uses parameters and notations detailed in tables 1 and 2. Birth and death of rats were excluded from the model, as well as the notion of slowly decreasing rat immunity. Birth and death of humans are not thought to be relevant at the time scale considered, but were nevertheless included in the model (at equal rates *b*_H_ and *d*_H_) in order to account for non-plague death recorded in the data.

**Table 1.** States of the epidemiological compartmental model.

	rat states
*S*_R_	number of susceptible rats
*I*_R_	number of infected rats
*Q*	number of infectious rat carcasses
	human states
*S*_H_	number of susceptible humans
*I*_H_	number of infected humans
*D*_H_	number of dead humans

**Table 2.** Notations and parameters used in the epidemiological model.

	rat parameters	
*T*_R_	number of live rats	*S*_R_ + *I*_R_
*K*_R_	initial size of rat population	250 000
*p*_0_	proportion of rats initially infected	estimated
*β*_R_	transmission rate from rat carcasses to susceptible rats	estimated
*ρ*	rat carcass infectivity range	estimated
*γ*_R_	death rate of infected rats	1/18 per day
*δ*_R_	rate of loss of infectiousness of rat carcasses	estimated
	human parameters	
*N*_H_	initial human population size	250 000
*d*_H_	human non-plague death rate	estimated
*b*_H_	human birth rate	*d*_H_
*β*_H_	transmission rate from rat carcasses to susceptible humans	estimated
*β*_I_	interhuman transmission rate	estimated
*γ*_H_	rate of death of infected humans	1/18 per day
*g*_H_	probability of human survival from plague	0.1

Infectious fleas were not explicitly modelled, but were replaced with the concept of infectious rat carcasses (state *Q*) which result from the death of infected rats and can cause infection in both susceptible rats and susceptible humans until infectivity is lost (rate *δ*_R_ which corresponds to the death of fleas). The only process that was added relative to the previous model is the transmission of plague from humans to humans at rate *β*_I_ (either directly or via vectors specific to humans) in order to investigate the relative role of this mode of transmission. A deterministic version of the resulting model is described by the following set of six ordinary differential equations:2.1
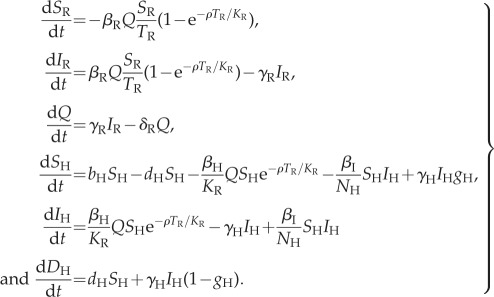


### 2.3. Stochastic simulation of model

We used a stochastic version of the deterministic compartmental model described in equation (2.1). The process is initialized on 1 January 1801 with a fully susceptible human population of size *N*_H_ and a rat population of size *K*_R_ with a proportion *p*_0_ of infected animals and the remainder being susceptible. The compartmental variables are, therefore, initialized as follows:2.2
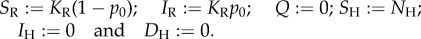


Simulation can then proceed by repeating the following two-step process for every day. First a vector *d*_1.8_ of transition variables is drawn from the following distributions:2.3
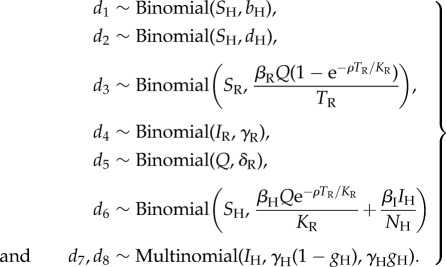


Second the compartmental variables are updated from one day to the next as follows:2.4
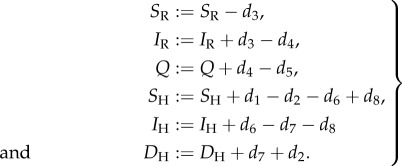


### 2.4. Bayesian inference

We considered that at the end of each day a number of human deaths is recorded which is Poisson distributed with parameter equal to 0.8 times the number of humans who died (i.e. entered state *D*_H_) on that day. Based on these observations, we want to infer the value of the seven parameters *p*_0_, *β*_R_, *ρ*, *δ*_R_, *d*_H_ = *b*_H_, *β*_H_ and *β*_I_.

The likelihood of the observed data under our model cannot be calculated analytically, but it can be approximated using a sequential Monte Carlo method [39] otherwise known as a particle filter. This approximation can be incorporated within a Markov chain Monte Carlo method to sample from the correct posterior distribution of parameters, and the resulting algorithm is known as a particle Markov chain Monte Carlo (pMCMC) [40]. Performing such Bayesian inference requires to specify prior distributions on the parameters, which were chosen to be highly uninformative: the prior for *p*_0_ was Uniform from 0 to 1 (i.e. the full range of possible values), whereas the prior for the other six parameters was Uniform from 0 to ∞, which is an improper distribution but does not lead to improper posterior distributions.

We implemented our model using the R package pomp [41] which includes the functionality to perform Bayesian inference using a pMCMC algorithm. We used 5000 particles in the particle filter algorithm which was sufficient to robustly estimate the likelihood. The pMCMC included 1 × 10^6^ iterations which were discarded as burnin, and a further 1 × 10^7^ iterations sampled every 100 iterations. Four separate chains were run, compared using the R package coda [42] and found to be in good agreement based on the multivariate version of the Gelman–Rubin diagnostic [43,44] which was lower than 1.1 for all inferred parameters. The samples from the four chains were then combined for maximum robustness. The effective sample size of the combined results was greater than 200 for all inferred parameters.

## 3. Results

### 3.1. Non-parametric exploratory data analysis

**Figure 1.**
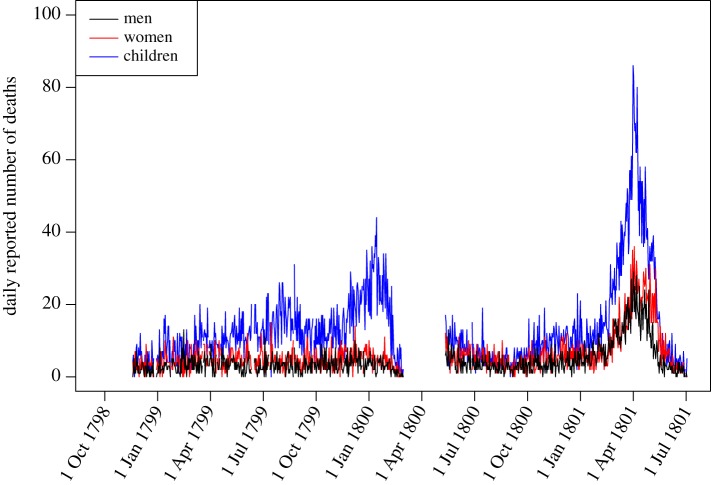
Daily reported number of deaths among men, women and children living in Cairo at the time of the French expedition. (Online version in colour.)

The French expedition into Egypt landed in Alexandria on 1 July 1798 and arrived in Cairo on 22 July, taking headquarters there until its departure in July 1801. For most of that period, namely between 19 November 1798 until 4 July 1801, the medical officers of the expedition recorded daily the number of deaths reported to have occurred among men, women and children living in Cairo. These data were published as a table in a book by the chief medical officer Desgenettes [30] which is summarized in figure 1 and the full curated data are reproduced in electronic supplementary material, table S1. A gap in the data in the Spring of 1800 was previously attributed incorrectly to the Syrian expedition of 1799 [35] but was in fact due to a revolt in Cairo at the time of the battle of Heliopolis. In the winter of 1799–1800, a significant increase in the number of deaths is seen among children only, which was caused by an epidemic of smallpox [30,32]. The number of daily deaths recorded between 1 October 1799 and 1 April 1800 is indeed significantly higher compared with the same period a year before in children (Wilcoxon–Mann–Whitney (WMW) test, *p* < 0.0001) but not in men (WMW test, *p* = 0.12) or women (WMW, *p* = 0.10). In the beginning of 1801, a large increase occurred in the number of deaths of men, women and children which was caused beyond doubt by an outbreak of bubonic plague [30–36]. In the first six months of 1801, the number of reported deaths is higher than in the same period of 1799 for men (WMW test, *p* < 0.0001), women (WMW test, *p* < 0.0001) and children (WMW test, *p* < 0.0001). This increase is of the same magnitude in children and adults (Fisher's exact test, *p* = 0.27) and is slightly higher in men than in women (Fisher's exact test, *p* = 0.03). Comparison between the first six months of 1799 and 1801 suggests that the Cairo 1801 plague outbreak was responsible for the recording of approximately 5000 human deaths.

After exclusion of 1801 due to the plague outbreak, the mean number of reported deaths was 18.81 per day or 6866 per year. The exact size of the population of Cairo at the time is unknown, but based on observations made by scientific members of the French expedition was estimated to be around 250 000 inhabitants [23,35,45]. Dividing this estimate of the population size by the mean number of annual deaths results in an estimated life expectancy at birth of 36.4 years. However, life expectancy was previously estimated to be around 28.5 years in 1800 globally [46]. In Europe where life expectancy more than doubled over the nineteenth and twentieth century, an average of 36 years was reached only in the 1850s [46]. This suggests that the number of deaths in Cairo was underreported by the medical officers, as indeed would be the case since they recorded only deaths that were brought to their attention. Considering that only 80% of deaths were reported leads to a more plausible estimate of the life expectancy of around 29.1 years.

### 3.2. Mathematical model of bubonic plague outbreak

**Figure 2.**
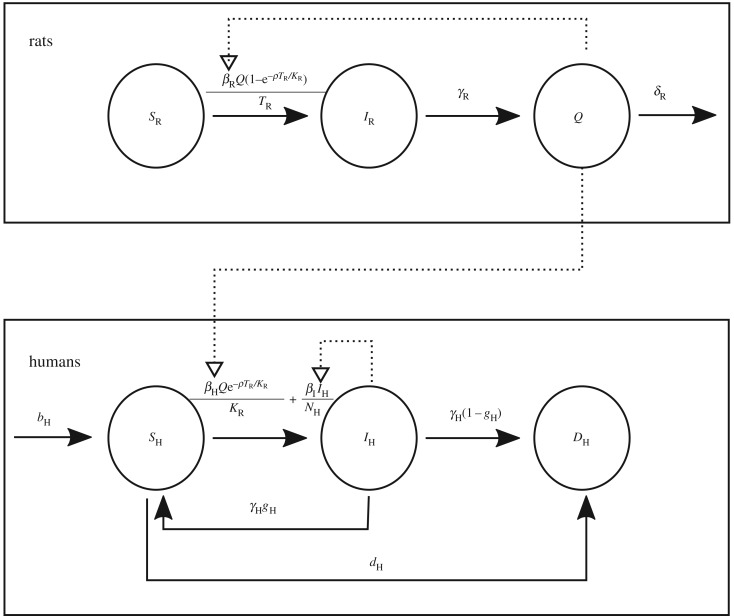
Compartmental model used for analysis. The three rat states are shown at the top and the three human states are shown at the bottom. Solid arrows represent probabilistic flow from one state to another, with rate indicated in the labels directly above. Dotted arrows represent the forces of infection exerted by infectious rat carcasses and infectious humans.

A model of bubonic plague has previously been proposed to describe the long-term dynamics of this disease [37,38]. We adapted this model to the study of outbreaks of the disease on a short time scale, and simplified it so that fleas are not explicitly represented. Our model is illustrated in figure 2 and all notations are summarized in tables 1 and 2. We first consider the population of rats, with initial size denoted *K*_R_. Most rats are initially susceptible (compartment *S*_R_) although a small proportion *p*_0_ are infected (compartment *I*_R_). Infected rats die at rate *γ*_R_ upon which they become an infectious rat carcass (compartment *Q*) which becomes non-infectious at rate *δ*_R_. The number of rats alive at a certain time is denoted *T*_R_ = *S*_R_ + *I*_R_. Meanwhile, the human population starts fully susceptible (compartment *S*_H_) and with size *N*_H_. Humans give birth at rate *b*_H_ and die (compartment *D*_H_) of non-plague causes at rate *d*_H_, and we assume *b*_H_ = *d*_H_ so that the population size would be on average constant without plague, over time scale relevant to outbreak. Infectious rat carcasses can make humans become infected (compartment *I*_H_), and the infection is resolved at rate *γ*_H_, upon which either death happens with probability 1 − *g*_H_ or recovery happens with probability *g*_H_. The two rates of infection from a rat carcass to a healthy rat or human are, respectively, equal to *β*_R_(1−exp(−*ρT*_R_/*K*_R_))/*T*_R_ and *β*_H_exp(−*ρT*_R_/*K*_R_)/*K*_R_ by analogy with the previous model [37,38] (cf. Material and methods). We also consider transmission from infected humans to susceptible humans happening at rate *β*_I_/*N*_H_ to account for the possibility of transmission via human ectoparasites [47] or pneumonic transmission [19]. The number of human deaths reported at the end of each day is assumed to be Poisson-distributed with mean equal to 80% of the actual number of deaths due to plague or non-plague causes, that is the number of transitions into the *D*_H_ compartment (figure 2).

The initial size of the rat population was set equal to the size of the human population, i.e. *K*_R_ = 250 000, as in a previous plague modelling study [48]. This choice is arbitrary as there is a complete lack of data available concerning the size of the rat population living in Cairo in 1801. However, the model was designed such that the value of this parameter does not affect the results. As long as the rat population size is large, it behaves almost deterministically, with the numbers of rats in states *S*_R_, *I*_R_ and *Q* being proportional to the chosen value of *K*_R_, and the parametrization was chosen so that only the proportions of rats currently in each state matters rather than actual number. Simulations confirm that multiplying or dividing *K*_R_ by 10 does not indeed change the dynamics of infection and that all other parameters are scale-free (electronic supplementary material, figure S1). The probability of human survival from plague was assumed to be equal to *g*_H_ = 0.1 which is the same value as used previously [38] and is also the value reported during the expedition into Syria that took place in 1799 [49]. The death rate of infected rats was set equal to 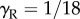 per day as in previous studies [37,38]. The death or recovery rate of infected humans was assumed to be 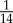 per day in a previous study [38], but this seems unrealistically low compared with clinical descriptions of bubonic plague disease progression [19]. Here we defined 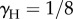 per day in accordance with the results of a previous modelling study [18]. We note, however, that if transmission is mostly driven by rats, *γ*_H_ has little effect of the disease dynamics. All other parameters described above, namely *p*_0_, *β*_R_, *ρ*, *δ*_R_, *β*_H_, *β*_I_, *d*_H_ and *b*_H_, were estimated from the data.

### 3.3. Estimation of the model parameters

**Figure 3.**
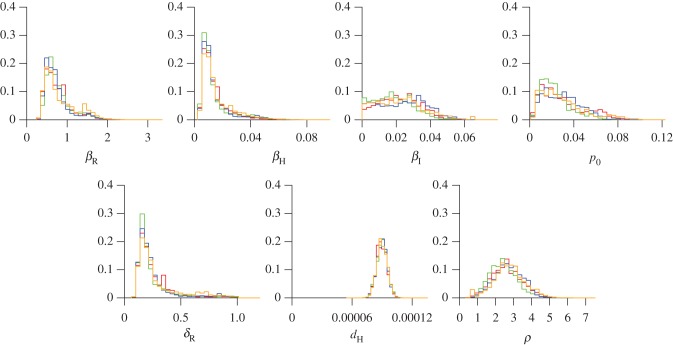
Posterior distributions of the seven estimated parameters, based on four separate pMCMC runs shown in four different colours. (Online version in colour.)

We applied the model of bubonic plague outbreak described above to the number of deaths reported in Cairo in 1801. Bayesian inference was implemented using a pMCMC [40], resulting in the posterior distribution of parameters shown in figure 3. For each parameter, we report the mean posterior estimate and 95% CI in square brackets. The parameter *d*_H_ represents the rate of human death for causes other than the plague outbreak and is estimated to be 8.98 × 10^−5^ [7.95 × 10^−5^;10 × 10^−4^]. This implies a life expectancy of 30.5 [27.3;34.5] years. This is comparable with the mortality rate observed in Cairo in 1799 and 1800 before the outbreak started (figure 1), which we estimated above to correspond to a life expectancy of 29.1 years. This consistency suggests that the model has successfully disentangled mortality caused by the plague outbreak and other causes.

The parameter *p*_0_ is the proportion of rats infected at the beginning of 1801 and is equal to 0.0279 [0.0052;0.0706]. This is small enough to justify the fact that we did not consider the rat infection dynamics prior to the beginning of 1801. This estimate of *p*_0_ implies that the plague epidemic started in the rat population towards the end of 1800, but would not at that time have had an effect on the human population as there was still a large majority of unaffected susceptible rats. The parameter *δ*_R_ represents the rate at which rat carcasses lose their infectivity and is 0.267 [0.116;0.845]. This means that on average rat carcasses remain infectious for 3.7 days [1.2;8.6]. This is in good agreement with experiments carried at the beginning of the twentieth century in Egypt which found that unfed rat fleas (*Xenopsylla cheopis*) survived on average approximately 4 days in January, February and March when temperatures are relatively low [27]. Previous studies [37,38] assumed a flea death rate of *d*_F_ = 10 per year implying a free flea average lifetime of 36.5 days, which would be unrealistically high for the Egyptian outbreak under study here.

The parameter *ρ* represents the rat carcass infectivity range and was estimated to be 2.63 [1.10;4.33]. This parameter is equivalent to *aK*_R_ in the previous published formulation [37,38] which showed that a human outbreak is possible as long as 0.5 < *ρ* < 20. A rat outbreak is only possible if the basic reproduction number for rats *R*_0_ = *β*_R_(1 − e^−*ρ*^)/*δ*_R_ is greater than 1. The parameter *β*_R_ was estimated to be 0.77 [0.39;1.66], and combining this with the previously mentioned estimates of *ρ* and *δ*_R_ leads to an estimated value of *R*_0_ of 2.85 [1.82;3.95]. Our estimates of the parameters *ρ*, *β*_R_ and *δ*_R_ are therefore compatible with the bubonic outbreak taking place in both the rat and human population as indeed would have been the case. Our estimate of the basic reproduction number for rats is very close to the previously assumed value of *R*_0_ = 3 [37,38]. The parameter *ρ* also determines the relative infectivity of rat carcasses on the human population, and the estimated value implies that they cause exp(*ρ*) = 13.9 [3.0;75.9] times more human cases when the rat population is fully depleted compared with when it is fully unaffected.

The parameter *β*_H_ represents the infectivity of rat carcasses to humans once the rat population has been fully depleted and was estimated to be equal to 1.45 × 10^−2^ [4.91 × 10^−3^;4.57 × 10^−2^]. The ratio *β*_H_/*β*_R_ represents the ratio of rates of transmission from rat carcasses to susceptible humans and rats, which given the estimated values of *β*_H_ and *β*_R_ was equal to 1.87 × 10^−2^ [6.00 × 10^−3^;3.87 × 10^−2^]. The bite of an infectious flea is therefore approximately 50 times more likely to cause an infection in a rat than in a human. The parameter *β*_I_ represents the transmission rate from humans to humans and was equal to 2.15 × 10^−2^ [1.13 × 10^−3^;4.78 × 10^−2^]. The parameters *β*_H_ and *β*_I_ were therefore roughly equal, indicating that after the complete collapse of the rat population, the force of infection to susceptible humans caused by an infectious rat carcass or an infectious human would have been comparable. However, the number *Q* of infectious rat carcasses is always at least two orders of magnitude higher than the number *I*_H_ of infectious humans, so that the contribution of interhuman transmission to the total human death toll is actually small. Another way of interpreting the value of *β*_I_ is to consider that if transmission occurred only between humans, the basic reproduction number for humans would be *R*_0_ = *β*_I_*N*_H_/(*γ*_H_*N*_H_) = *β*_I_/*γ*_H_ which is equal to 0.172 [0.090;0.382] and therefore much smaller than 1 so that an epidemic could never take place in these conditions.

**Figure 4.**
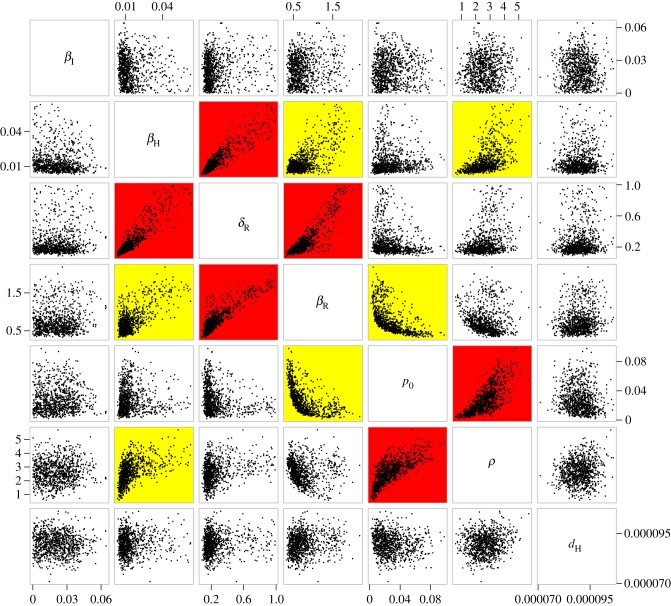
Scatter plots illustrating the relationships between all pairs of estimated parameters. A red background indicates a correlation higher than 0.7, a yellow background indicates a correlation between 0.3 and 0.7 and a white background indicates a correlation lower than 0.3. The scatter plots are based on a thousand samples from the posterior distribution. (Online version in colour.)

The posterior relationships between the seven parameters are illustrated in figure 4. Only the two parameters *β*_I_ and *d*_H_ did not show any strong correlation with other parameters, as would be expected from the fact that they correspond to death from non-plague causes and human-to-human plague transmission, both of which are unrelated to the remainder of the parameters which concern rat transmission dynamics. The strongest correlations were found between *δ*_R_ and *β*_H_ (0.92) and between *δ*_R_ and *β*_R_ (0.87), which corresponds to the relative trade-off between duration (1/*δ*_R_) and level of infectivity (*β*_R_ and *β*_H_) of the rat carcasses. The third strongest correlation was found between *ρ* and *p*_0_ (0.73), and corresponds to the timing of the epidemic in humans, which started approximately 50 days into 1801 (figure 1). If the proportion *p*_0_ of infected rats at the start of 1801 was low then the flea infectivity range *ρ* has to be low so that the infection spreads to humans before the rat population is fully depleted. Conversely, if the proportion of infected rats *p*_0_ was initially high then *ρ* has to be high so that infection only spreads to humans after most rats are dead. But if only one of these two parameters was high, the number of human deaths would have started to increase earlier than it did.

### 3.4. Analysis of posterior predictive simulations

**Figure 5.**
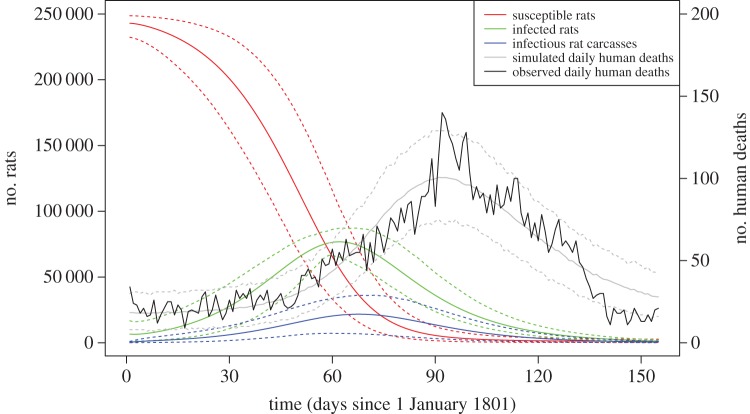
Simulation of the model using 1000 values from the posterior sample. Solid lines indicate the mean value across the 1000 simulations, while the dotted lines indicate the range. (Online version in colour.)

We performed an analysis of posterior predictive simulations in order to provide a Bayesian assessment of model fitness [50]. We simulated 1000 outbreaks each of which started on 1 January 1801 using the same model as used for inference and with parameters drawn from the posterior sample (figure 5). The number of human deaths recorded to have happened daily in these simulations can be compared with the actual number of deaths reported in Cairo in 1801 (figure 5). Overall there is a good agreement between the simulated and real data, with the real data being almost always within the 95% confidence interval of the posterior predictive distribution, which suggests a good fit of the model to the data [50]. The only exception concerns the very end of the timeframe, from day 140, where the number of recorded deaths is slightly but consistently below that expected under the model. The number of human deaths recorded during that time is even lower than before the start of the outbreak, which could be as a result of a small fraction of the population having fled from the outbreak, or because the French medical officers recorded a smaller proportion of deaths at that time which directly precedes the end of the expedition.

The fate of the rat population in these posterior predictive simulations is also shown in figure 5. At the start of 1801, the vast majority of rats were susceptible (as defined by the *p*_0_ parameter). In the first few days, the infection spread in the rat population only slowly, but the epidemic progressively gathered momentum, with the majority of the rat population being infected by day 50. The number of human deaths up to that point was, however, small due to the preference of fleas to infest rats rather than humans, and the still large proportion of susceptible rats available. The number of reported human deaths caused by plague, however, increased steadily from day 50 up to approximately day 90 where it peaks. The number of infectious rat carcasses peaks beforehand, around day 70, but at that point approximately 10% of the original rat population was still susceptible and therefore protecting the human population. Between day 70 and 90 this proportion drops down to almost nothing, so that the incidence in the human population continues to increase. After day 90 the number of infectious rat carcasses declined, explaining the decline in the number of human deaths which went back down to pre-outbreak levels around or soon after day 150. The number of humans infected by plague in these simulations was on average 7096 [6560;7622]. The number of human plague infections caused by human-to-human transmission was on average 1265 [78;2899] or 18% [1%;41%]. Among all cases of plague, 696 [626;774] individuals recovered and 6282 [5806;6758] individuals died as a result of the infection. Among the humans who were killed by plague, the number of recorded deaths was on average 5027 [4651;5405], which fits with our previous calculation based on a comparison of 1801 with 1799 and 1800.

## 4. Discussion

From a historical point of view, the outbreak of bubonic plague that took place in Cairo in 1801 was by no means unique, and indeed there would have been many similar events in Egypt and elsewhere in the Middle East at the turn of the nineteenth century [26]. However, what makes this outbreak worthy of scientific attention is the collection and recording of epidemiological data by the the medical doctors who took part in the Napoleonic French expedition into Egypt. Although these data are of course imperfect, it enabled us to infer many properties of this outbreak, in contrast with, for example, the previous large epidemic that occurred in Cairo in 1791 for which only anecdotical information survives [25].

We estimated that approximately 5000 people died of plague in 1801 in Cairo which represents about 2% of the 250 000 strong population living at the time in Cairo. This mortality is relatively low compared with some of the famous outbreaks of bubonic plague that occurred previously towards the end of the second pandemic, for example, approximately 50% in Genoa and Naples in 1656, approximately 20% in London in 1665 and approximately 40% in Marseilles in 1720 [12,16,17]. The third plague pandemic that started in the 1890s in China caused lower mortality rates, presumably as a result of improving housing conditions and public health practices [12]. For example, when Hong Kong was hit by plague in 1894, it had a population size of approximately 210 000 Chinese, of whom 1–3% died of the disease [51]. It is during this epidemic that Alexandre Yersin discovered the bacterial agent causing bubonic plague and its transmission routes, which would have important consequences in the fight against plague and other infectious diseases [52].

We found that most human cases of plague during the Cairo 1801 outbreak were caused by transmission from the rat population. Our point estimate for this proportion was 82% but the confidence interval reached almost to 100%, suggesting that there is in fact no evidence for human-to-human transmission during this outbreak. Again this fits well with observations of the central role played by zoonotic infection during the third bubonic plague pandemic (with the exclusion of pneumonic plague) as opposed to the second pandemic during which human-to-human transmission is increasingly thought to be important [53] and was estimated to be responsible for three quarters of cases during the 1665 outbreak in Eyam [18]. The outbreak that took place in Cairo in 1801 between the second and third pandemics therefore seems to share more of the typical epidemiological characteristics of the third pandemic, that is with a relatively low mortality, a clear role of rodent-to-human infection and little evidence for human-to-human transmission. It should, however, be noted that the differences between the two pandemics are not clearly established, and that each outbreak may in fact have unique properties due to local conditions. Consequently, it is not possible to draw conclusions based on this single outbreak concerning the wider epidemiology of plague ravaging the Middle East at that time.

The statistical model we described was adapted from previous work [37,38] with three aims in mind: to minimize the number of parameters that need to be estimated, to focus on dynamics taking place on a short time scale and to allow the estimation of balance between zoonotic and interhuman transmission. The design of this model was not based on any specific consideration about the Cairo outbreak, and should therefore be applicable to other outbreaks of bubonic plague, along with the pMCMC method we developed to perform Bayesian inference. It seems unlikely that enough data survive concerning the first pandemic to perform any such analysis, but the comparative application of our model and inference procedure to multiple outbreaks of the second and third pandemics of bubonic plague should reveal a clearer picture of the complex epidemiology of this important infectious disease.

## Supplementary Material

Figure S1

## Supplementary Material

Table S1

## Data accessibility

All data used in this study are available in electronic supplementary material, table S1.
