# Supplementary figures and images for: Model-based analysis of an outbreak of bubonic plague in Cairo in 1801
Source: J R Soc Interface. 2017 Jun 21;14(131):20170160. doi: 10.1098/rsif.2017.0160 (PMC5493801; doi:10.1098/rsif.2017.0160)

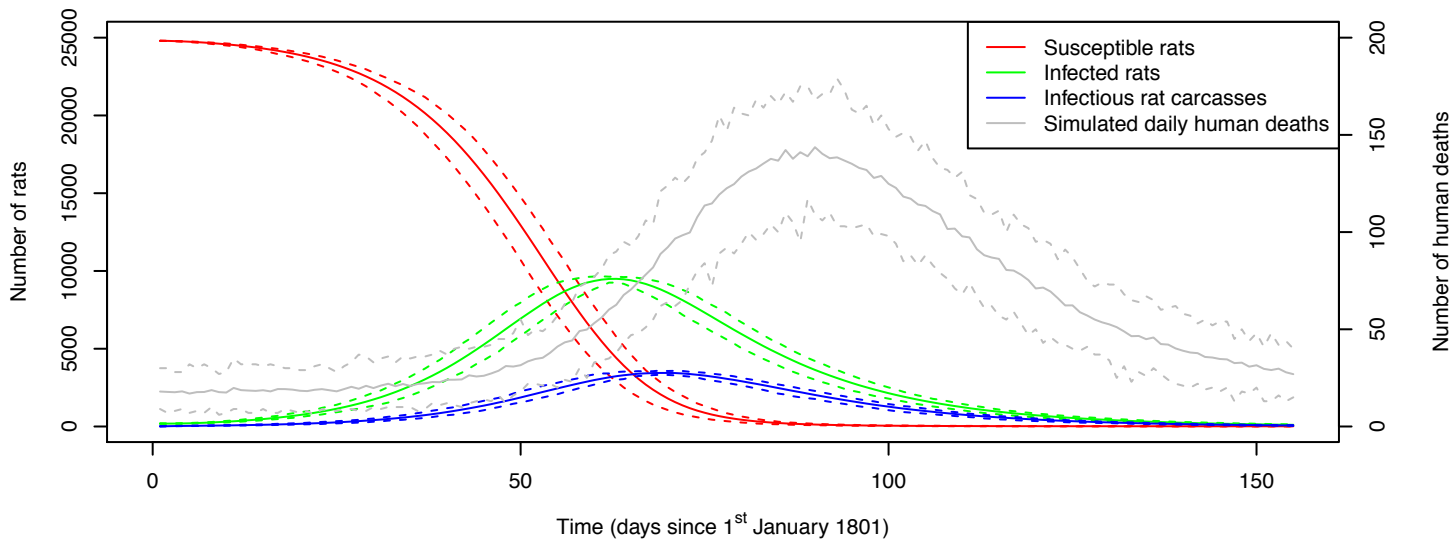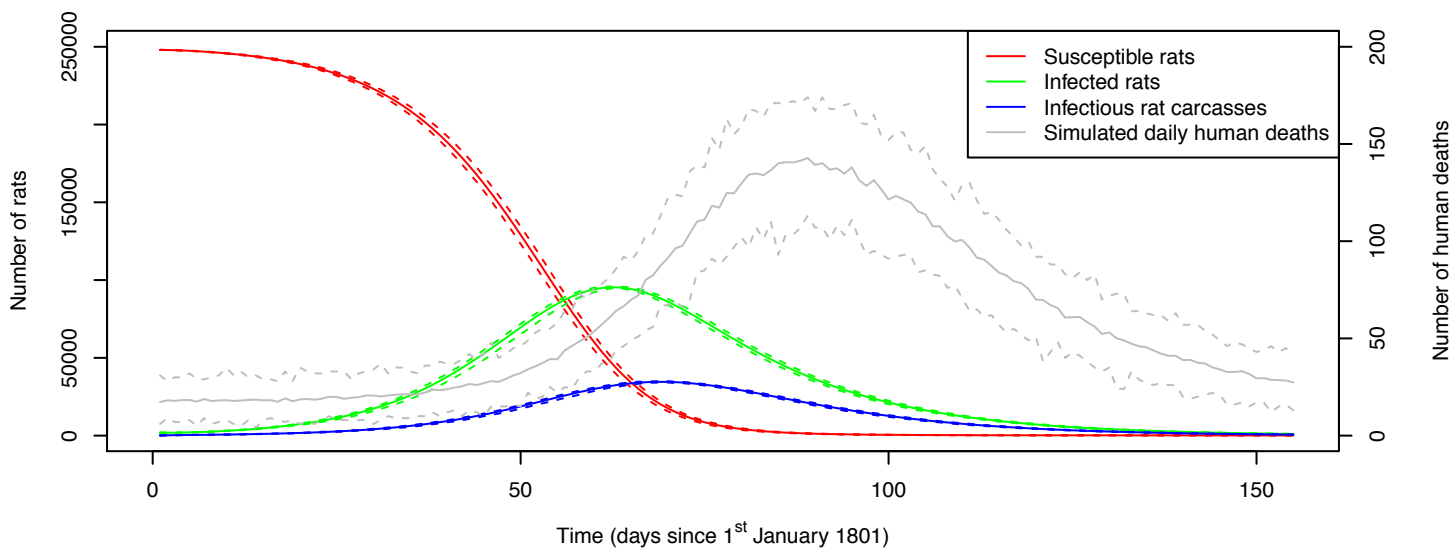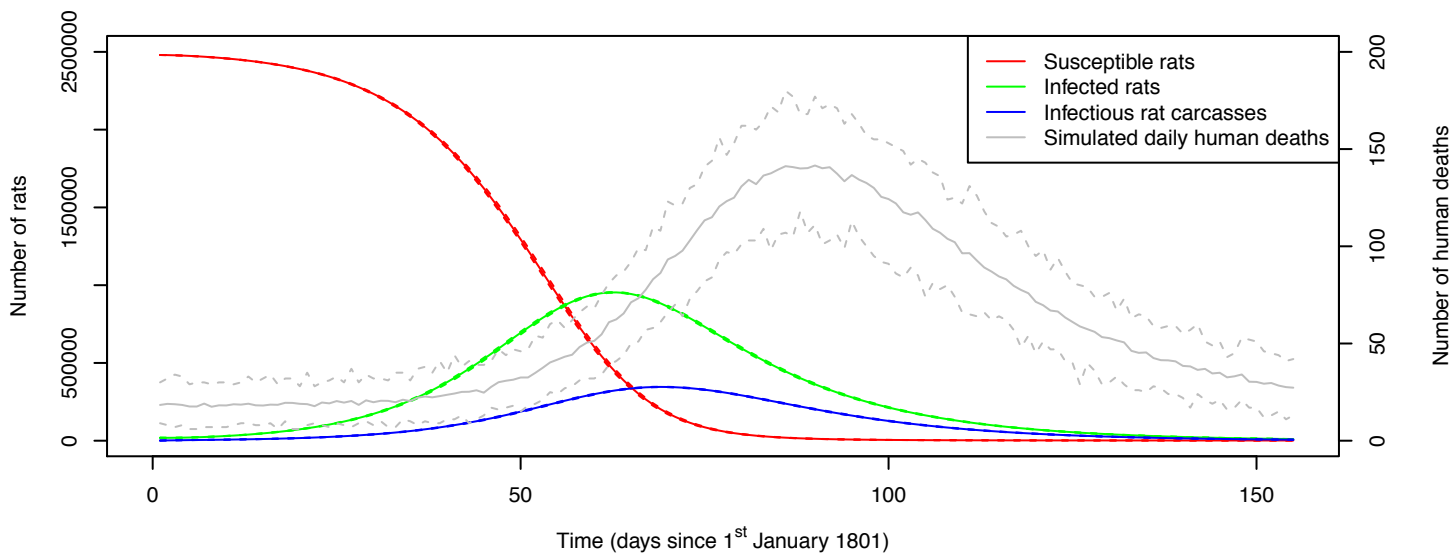

Supplement: Figure S1 [file rsif20170160supp1.pdf]
